# Photoresponse of supramolecular self-assembled networks on graphene–diamond interfaces

**DOI:** 10.1038/ncomms10700

**Published:** 2016-02-25

**Authors:** Sarah Wieghold, Juan Li, Patrick Simon, Maximilian Krause, Yuri Avlasevich, Chen Li, Jose A. Garrido, Ueli Heiz, Paolo Samorì, Klaus Müllen, Friedrich Esch, Johannes V. Barth, Carlos-Andres Palma

**Affiliations:** 1 Chemie-Department, Technische Universität München, Lichtenbergstraße 4, Garching 85748, Germany; 2 Catalysis Research Center, Technische Universität München, Ernst-Otto-Fischer-Straße 1, Garching 85748, Germany; 3 Physik-Department, Technische Universität München, James-Franck-Strasse 1, Garching 85748, Germany; 4 Walter Schottky Institut, Technische Universität München, Am Coulombwall 4, Garching 85748, Germany; 5 Max Planck Institute for Polymer Research, Ackermannweg 10, Mainz 55128, Germany; 6 ISIS & icFRC, Université de Strasbourg & CNRS, 8 allée Gaspard Monge, Strasbourg 67000, France

## Abstract

Nature employs self-assembly to fabricate the most complex molecularly precise machinery known to man. Heteromolecular, two-dimensional self-assembled networks provide a route to spatially organize different building blocks relative to each other, enabling synthetic molecularly precise fabrication. Here we demonstrate optoelectronic function in a near-to-monolayer molecular architecture approaching atomically defined spatial disposition of all components. The active layer consists of a self-assembled terrylene-based dye, forming a bicomponent supramolecular network with melamine. The assembly at the graphene-diamond interface shows an absorption maximum at 740 nm whereby the photoresponse can be measured with a gallium counter electrode. We find photocurrents of 0.5 nA and open-circuit voltages of 270 mV employing 19 mW cm^−2^ irradiation intensities at 710 nm. With an *ex situ* calculated contact area of 9.9 × 10^2^ μm^2^, an incident photon to current efficiency of 0.6% at 710 nm is estimated, opening up intriguing possibilities in bottom-up optoelectronic device fabrication with molecular resolution.

## Abstract (web summary)

Two-dimensional, self-assembled heteromolecular networks often lack functionality. Here the authors study the photoresponse of self-assembled heteromolecular networks, while controlling their positions and interfaces at an atomic level, suggesting bottom-up assembly of optoelectronics devices.

## Graphical abstract

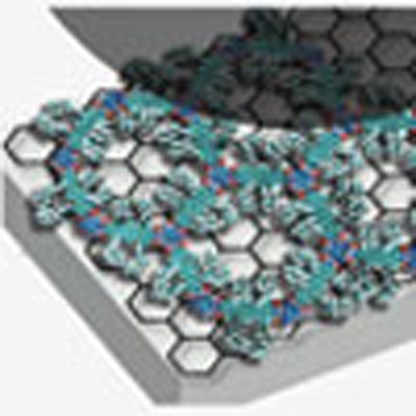

Elemental crystals, interfaces and (macro)molecules have become integral parts of modern semiconductor devices which are shaping twenty-first century technology. Thus far, the first generation of organic semiconductor devices, for example, light-emitting diodes^1^ and photovoltaics^2^, are based on (macro)molecules processed with a wide range of methods in an effort to make them compatible with the capabilities of the semiconductor industry. These methods consist mainly of thin film technologies, including spin-coating^3^, sublimation^1^, printing^4^, crystallization^5^ and self-assembly^6^,^7^,^8^ fabrication procedures or combinations thereof^9^. However, the aforementioned strategies have been often centred around the fabrication and the tuning of the properties of the (macro)molecular active components in thin films^10^. Consequently, spatial orientation of both the active layer's components and their interfaces are not usually known with atomic precision. Thus, *a posteriori* characterization techniques are required and the final absolute atomic-scale spatial constitution of the device as a whole is rarely reproducible. Although an atomically precise layout of the constituting components is not always required for a device's function, it may be critical for its optimization^11^. For instance, sensitizing interfaces with molecules was early recognized^12^ as efficient means to photovoltaic charge generation. By optimizing the sensitizer surface area, this strategy became technologically viable^13^,^14^. Similarly, by improving chemical precision over donor and acceptor polymers^15^, organic solar cell efficiencies grew rapidly^16^. Thus, it is clear that for a transition to a second generation of organic device engineering, their constituents must be fabricated not only with high interfacial and chemical control but also with exquisite spatio-temporal heteromolecular precision, where the absolute location of different molecular components is mastered and precisely known *a priori*. So far, device elements approaching such molecular precision employ single-molecule configurations^17^,^18^, which are not yet ready to be implemented for large-area technological applications. One strategy for large-area, artificial molecularly precise device fabrication is to grow architectures from the bottom-up^19^, at interfaces with solutions^20^ or under vacuum. Supramolecular hydrogen bonded^21^,^22^, metal-organic^23^,^24^ or covalent^25^,^26^,^27^ multi-component surface-confined networks provide a route to precisely organize different building blocks relative to each other in two dimensions (2D). These 2D surface assemblies can be engineered^28^,^29^ with increasing level of prediction^30^,^31^ to afford device functionalities ontop of specific substrates. In addition, 2D networks can act as templates for the growth of three-dimensional networks^32^,^33^, paving the road towards vertical heteromolecular control via monolayer-by-monolayer growth. Such architectures may present precise interpenetrated morphologies^34^ with ideal nanoporous and columnar order, which have long been considered optimal configurations for organic solar cells^11^,^35^.

Here we demonstrate the photoresponse of a bicomponent supramolecular network (Fig. 1a) on transparent, graphene-passivated H-C(100) diamond (GHD) and employing a gallium droplet as a counter electrode (Fig. 1b). The network is built with a chromophore, consisting of a terrylene diimide (TDI) derivative (**1**) and melamine (**2**). After initial molecular characterization by means of scanning tunnelling microscopy, we show generated photocurrents of (0.5±0.2) nA and photovoltages of (270±120) mV at 19 mW cm^−2^ irradiation intensities at 710 nm (uncertainties are s.d. in tenths of measurements for different sample preparations). We find incident photon to electron efficiencies (IPCE) at 710 nm of (0.6±0.25)% when estimating the contact area *ex situ*, yielding photocurrent densities of (47±5) μA cm^−2^. Our work introduces bottom-up supramolecular network engineering on 2D materials for molecularly precise function with atomically defined interfaces.

## Results

### Chemical design and synthesis

**Figure 2:**
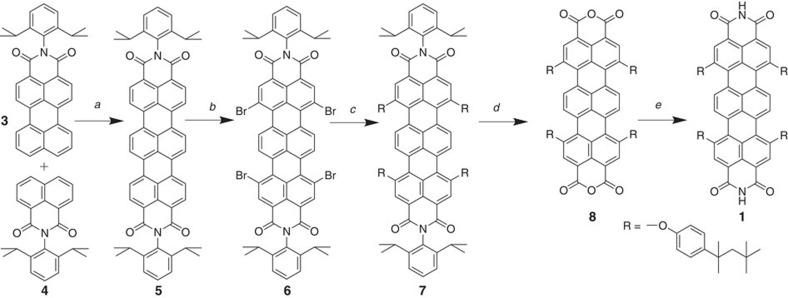
Synthesis of novel TDI (1). (a) Base-induced fusion of naphthaleneimide and peryleneimide: diazabicyclo[4.3.0]non-5-ene, *t*-BuONa, diglyme, 130 °C, 3 h, 42%; (b) tetrabromination of TDI: Br_2_, chloroform, reflux, 12 h, 75%; (c) phenoxylation of TDI: 4-(1,1,3,3-tetramethylbutyl)phenol, K_2_CO_3_, N-methylpyrrolidone, 80 °C, 8 h, 86%; (d) base-induced hydrolysis of bisimide into bis-anhydride: KOH, KF, 2-methyl-2-butanol, reflux, 53%; and (e) NH-imidization of terrylene bis-anhydride: ammonium acetate, propionic acid, reflux, 20%.

Heteromolecular recognition between tritopic melamine and complementary ditopic linkers via hydrogen bond formation was introduced as means to achieve hexagonal supramolecular architectures^21^,^36^. By proper chemical design, the complementary linker can be engineered^31^,^37^ for self-assembly with increasing level of predictability at the solid–liquid interface, thereby decreasing the content of poorly ordered and glassy phases. For instance, we have previously shown that peripheral substitution of the linker favours the formation of networks over tightly packed patterns^22^. Further, the linker can be imbued with functional properties. Rylene dyes are poly(peri-naphthalene)s^38^ that show a high photostability^39^, which make them preferred constituents for application in optoelectronic devices. Extending the *π*-system of the dye allows for tuning the optical properties and thus, shifting the absorption maximum to higher wavelengths. At the same time, rylenes are known to be amongst the most efficient organic absorbers^38^,^40^. Thus, rylenes are promising for the infrared regime with high transparency in the visible spectrum, for use in the next generation of facade and window building technology^41^. By introducing diimide terminations, the molecules acquire the supramolecular moiety necessary for triple hydrogen bond complementary recognition with the melamine cornerstone. Substituents at the bay position are used to improve the solubility^38^ and favour porous network formation. Thus a novel TDI, bearing NH groups in the imide structure, is synthesized according to Fig. 2 (see Supplementary Methods for details). The presence of NH groups makes the solubility poor and purification of the rylene dyes rather difficult. Therefore, we use bulky 2,6-diisopropylphenyl groups in the starting one-pot reaction of perylene monoimide **3** and naphthalene monoimide **4** to create the soluble compound **5**. After bromination and phenoxylation steps, tetra(*t*-octylphenoxy) substituted **7**, showing outstanding solubility^42^, can be used in the following. Hydrolysis of the imide groups under basic conditions, results in bis-anhydride **8** which is reacted with ammonium acetate to afford the target **1**. In both cases the presence of four voluminous *t*-octylphenoxy groups made purification and processing possible. Comparing with soluble perylene diimide (PDI) analogues, TDIs have higher absorption coefficients, which render TDI a better light-harvesting efficiency^43^. Moreover, the additional naphthalene unit in the TDI structure separates the tetraphenoxy groups and makes the TDI molecules less twisted^44^. Thus, improved planarity and increased absorptivity^38^,^40^ render the engineered TDI supramolecular assemblies advantageous over shorter PDI assemblies^21^,^45^.

### Scanning tunnelling microscopy characterization

**Figure 3:**
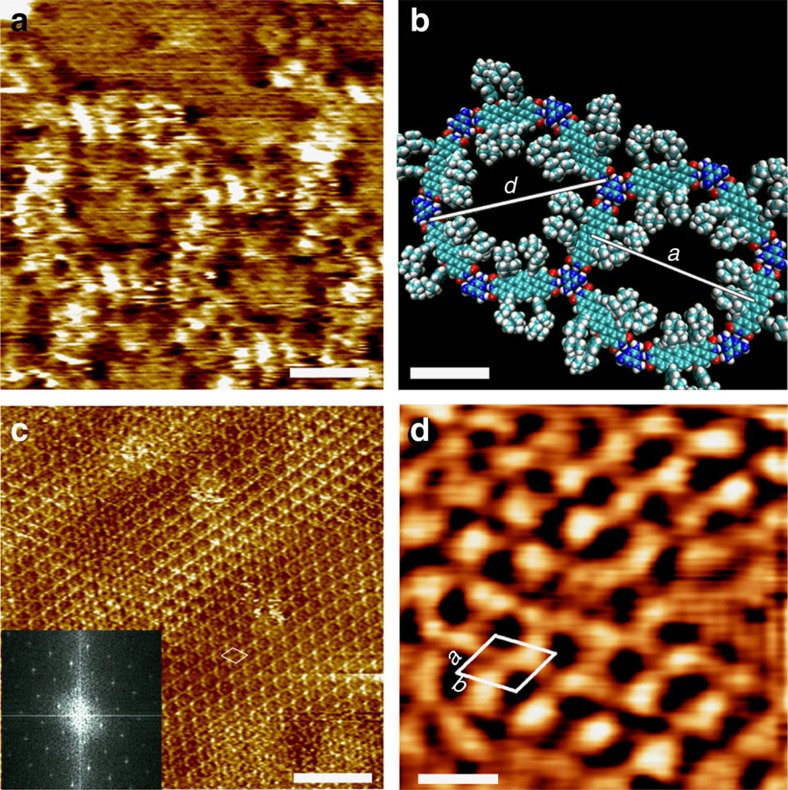
STM images showing the assembly of 1+2 on graphite and graphene-passivated H-C(100) diamond substrates. (**a**) STM constant current image of **1+2** (16 μM: 10 μM) and underlying HOPG interface. Scale bar, 2 nm (**b**). Molecular model minimized by the MMFF force field, the hexagonal size corresponding to the theoretical unit cell is *a*=*b*=4.2 nm and the pore diameter is *d*=4.6 nm. Scale bar, 2 nm (**c**). STM large-area constant height image (12 μM:8 μM). (inset) 2D fast Fourier transform showing the high crystallinity of the assembly on HOPG. Unit cell *a*=(4.1±0.2) nm, *b*=(4.3±0.2) nm and *a*,*b*=65±2° Scale bar 20 nm (**d**). Gaussian-filtered STM constant current image of **1+2** on GHD. Unit cell *a*=(3.9±0.2) nm and *b*=(4.0±0.2) nm and *a*,*b*=62±2°. Area 13.8 nm^2^. Tunneling parameters: average tunneling current (*I*_t_)=20 pA, sample voltage (*V*_t_)=300 mV. Scale bar, 5.5 nm.

The successful formation of highly regular 2D supramolecular networks between **1** and **2** has been investigated *ex situ* (on a model substrate) and *in situ* (in the device configuration) by means of scanning tunnelling microscopy. Self-assembly experiments involved applying a mixture of **1+2** in 1,2,4-trichlorobenzene (TCB) with 1–5% of dimethylsulfoxide (DMSO), first on highly ordered pyrolytic graphite (HOPG, Fig. 3a,c) and then on transparent platforms made by graphene transferred to hydrogenated H-C(100) diamond (GHD). In the scanning tunnelling microscope (STM) images, molecular features can be resolved. Figure 3a shows how the observed hexagonal supramolecular features perfectly match the expected chemical structure in Fig. 3b. The molecular geometry was optimized with the MMFF molecular force field. The 2D fast Fourier transform in Fig. 3c provides evidence for the regularity in the monolayer supported on HOPG. At GHD, only nanocrystalline hexagonal domains with sizes of tens of nanometres are monitored (Fig. 3d). Defects and impurities on GHD make the extended growth of crystalline bicomponent networks challenging, as discussed below. The experimental unit cell parameters for the network formation on GHD amount to *a*=(3.9±0.2) nm and *b*=(4.0±0.2) nm and *a*,*b*=62±1°, which are in perfect agreement with the values measured on HOPG.

### UV–visible measurements

**Figure 4:**
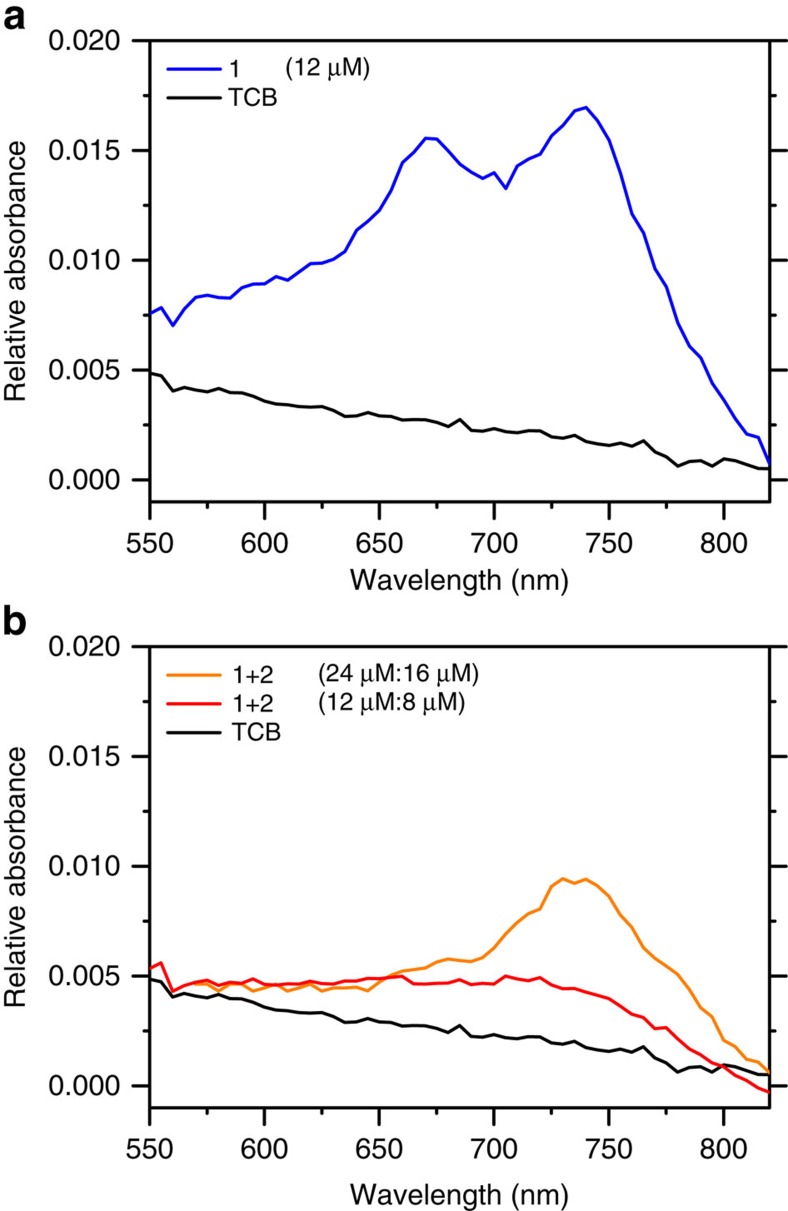
UV–vis spectroscopy on graphene-passivated H-C(100) diamond. (**a**) **1** (12 μM) and pure TCB baseline spectra. (**b**)**. 1+2** (12 μM:8 μM) and pure TCB baseline absorbance spectra. **1+2** (24 μM:16 μM) is also shown as additional evidence of strong absorbance reduction upon complexation with **2**.

With an atomically flat and transparent platform such as GHD, capable of supporting the bimolecular 2D self-assembly, optical spectral properties of crystalline supramolecular layers can now be investigated. Thus, UV–vis absorption measurements were performed to distinguish between changes in the spectrum of **1** upon hydrogen bond recognition with **2**. The absorption spectra of (5±1) μl of the pristine **1** in a 12 μM TCB solution and the pure TCB solvent on the diamond supported graphene surface are shown in Fig. 4a. Two distinct absorption peaks at 665 and 735 nm can be distinguished. This corresponds to a bathochromic shift of 46 and 66 nm with respect to UV–vis measurements in solution (Supplementary Fig. 1). Note that the TCB solvent used for the drop-casting shows no absorption in that wavelength range. The absorption peak-to-baseline signal of **1**, 0.012 at (735±2) nm (see Supplementary Fig. 1 for absolute absorption units), is indicative of approximately a monolayer of **1** when compared with the absorbance of monolayer perylene tetracarboxylic anhydride on graphene^46^, ∼0.007 at 702 nm. The molar attenuation coefficient of perylene tetracarboxylic anhydrides and PDIs^47^ of ∼5 × 10^4^ M^−1^ cm^−1^ is half the one of analogue TDI derivatives^48^, close to 1 × 10^5^ M^−1^ cm^−1^, at the respective absorbance maxima. When (5±1) μl solutions of **1** and **2** with a concentration ratio of 12 μM:8 μM are applied to the sample, a fivefold reduction of the signal of **1** is observed along with a bathochromic shift of the absorbance maximum to (740±5) nm (Fig. 4b, uncertainties are s.d. between four different preparations). At least a 1.5-fold reduction in the absorbance is expected when comparing the molecular density of molecules of **1** (0.31 nm^−1^) with that of molecules of **1** in the **1**+**2** network (0.21 nm^−1^). Incidentally, a fourfold reduction of the absorbance maximum is also prominent in *π*-stacks of perylenes^49^, in part due to specific surface reduction^50^,^51^. Because the reduction of the absorbance is not an effect of variations in the drop-cast solution volume or concentration (see Supplementary Fig. 1), we suggest it is the combined effect of a looser packing (increased unit cell) of **1+2** and its aforementioned *π*-stacking^33^.

### Photoresponse characteristics

**Figure 5:**
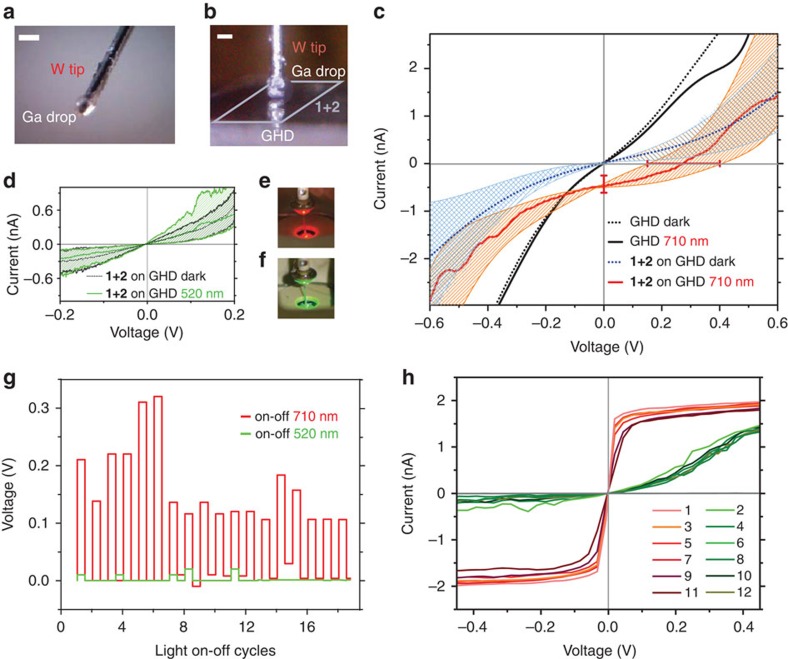
Photoresponse of a supramolecular network optoelectronic device element. (**a**). Tungsten STM tip with a gallium droplet. (**b**) Gallium electrode in contact with the sample before the tunneling spectroscopy measurements. (**c**). Current–voltage characteristics (contact regime see main text) of pristine GHD (black lines) and of **1+2** on GHD before (blue lines) and after (red lines) irradiation at *λ*=710 nm (**d**). Current–voltage characteristics before (black lines) and after (green lines) *λ*=520 nm photon irradiation. Approach parameters: *I*_t_=2 nA and *V*_t_=100 mV. Striped areas indicate the maximum and minimum currents observed in more than ten curves in a single junction, while error bars indicate the standard deviation for five junctions in three samples and are reported in the main text as *I*_SC_=(0.5±0.2) nA, *V*_oc_=(270±120) mV (**e**). Back illumination geometry with λ=710nm (**f**). Back illumination geometry with λ=520nm. (**g**). Open-circuit voltage for consecutive on-off irradiation cycles at 710 nm (red lines), followed by on–off cycles at *λ*=520 nm (green lines) of **1+2**. Every set of six consecutive cycles was recorded with different junction (sample, tip) preparations. The constant dark voltage background for each set was substracted (∼0.05 V). For stability, these studies were performed with functionalized eutectic gallium-indium electrodes, see Methods. (**h**) Scanning tunnelling spectroscopy (non-contact regime, see main text) for consecutive irradiation cycles 1–12 at *λ*=710 nm (red lines), followed by *λ*=520 nm (green lines), showing drastic changes assigned to electron tunneling through photoexcited states. Setpoint parameters: *I*_t_=1 nA, *V*_t_=300 mV. For all panels, data shown was neither filtered nor averaged. Scale bars, 250 μm.

To preserve pristine molecular interfaces aiming at molecular precision, a gallium droplet has been used to soft-contact the supramolecular network. Current–voltage (*IV*) measurements have been used to characterize the device element's photoresponse. The top contact was fabricated by letting a liquid gallium droplet cover a blunt tungsten tip and slowly cool at room temperature (Fig. 5a). Gallium alloys have been widely used as a replacement for mercury for creating macroscopic device contacts with molecular layers^52^,^53^. Our strategy consists of approaching the gallium-coated tip to the substrate and stabilizing it to a current setpoint of 2 nA at 100 mV, with the help of a modified STM. Contact and wetting of the GHD substrate by the gallium was inferred by fluorescence spectroscopy (see Methods and Supplementary Fig. 2) and by measuring the current during approach of the electrode to the surface (Supplementary Fig. 3). In addition, stepwise increase of the setpoint current shows stable semiconductor characteristics up to 100 nA (Supplementary Fig. 4). The large-area *IV* measurements were performed on the **1+2** on GHD and bare GHD substrates. The *IV* spectra of GHD (Fig. 5c, black dotted line) and **1+2** on GHD (Fig. 5c, blue dotted line) were recorded with a forward sweep of 200 mV s^−1^. For obtaining the photoresponse characteristics, the current was recorded under illumination by a red light-emitting diode (LED) (*λ*=710 nm) with a measured power of 19 mW cm^−2^. When bare GHD was employed, no photoresponse was observed (Fig. 5c, black line). Conversely, the illuminated *IV* curve of the 12 μM:8 μM **1+2** on GHD monolayer photoresponse exhibits characteristic features (Fig. 5c, red line). Under illumination, a finite current flows at zero bias voltage, the short-circuit current *I*_SC_. An average short-circuit current of *I*_SC_=(0.5±0.2) nA and open-circuit voltage of *V*_oc_=(270±120) mV were measured by illuminating the system with monochromatic light of 710 nm. Typical maximum and minimum values, from the average photoresponse characteristic, are also shown as shaded areas in Fig. 5c. In addition, *IV* curves were recorded by illuminating the **1+2** network on graphene with monochromatic light of 520 nm of 13mW cm^−2^ (Fig. 5d), where **1** does not absorb light. Indeed, no photocurrent was generated under 520 nm light irradiation conditions where TDI does not absorb. Figure 5e,f depict the back illumination geometries employed. Figure 5g shows the stability of the photovoltage generated by the junctions as a function of light on–off cycles (employing functionalized gallium tips, see Methods). The data corresponds to ∼25% of various measured junctions. In the remaining junctions, a different regime is observed, where a clear increase in the current with 710 nm irradiation occurs but neither open-circuit voltage nor short-circuit current are detected (Fig. 5h). Because current-distance spectroscopy reveals a clear exponential dependence of the current with the distance, these junctions do not physically contact the substrate. Hence, this non-contact regime is attributed to a photoexcitation effect, where additional tunnelling channels are opened upon light exposure. All in all the results show that the supramolecular network based on **1+2** molecules specifically generates a photoresponse at the designated wavelength. It is instructive to approximate the tunnelling contact area of the gallium droplet (∼250 μm diameter) to estimate photoconversion efficiencies. By *ex situ* contact junctions with insulating fluorescence dyes (see Methods and Supplementary Fig. 2), an area of (9.9±0.6) × 10^2^ μm^2^ can be estimated for the *in situ* experiment. This area is roughly 2% of the projected area under the gallium electrode (49 × 10^3^ μm^2^, using a radius of 125 μm). With such estimation, current densities of 10^−4^ A cm^−2^ at 0.5 V can be derived from the *IV* spectra. These current densities are comparable to those reported by contacting -S-C_16_H_33_ self-assembled monolayers-metal interfaces of similar contact areas^53^, indicating a soft-contact. Further, with the derived photocurrent density of *J*_SC_=(47±5) μA cm^−2^ a monochromatic IPCE of (0.6±0.25)% can be estimated at 710 nm, 19 mW cm^−2^ irradiation intensities.

## Discussion

We have fabricated surface-confined bicomponent assemblies on GHD based on a functional dye^38^ absorbing at 740 nm. The active layer ideally consists of a self-assembled TDI-based supramolecular nanoporous network exhibiting nanocrystalline hexagonal order. Shorter diimide-based molecules like naphtalenes have been found to stack in the third dimension through van der Waals face-to-face stacking^33^. Thus, the employed system presents an avenue towards molecularly precise three-dimensional devices. During the measurements, atomically flat and transparent all-carbon GHD served as the photoanode, while a gallium junction was used as top cathode electrode, respectively. For sake of maintaining molecular integrity at the interface, as observed by STM in Fig. 3, a soft gallium electrode is used as a contact. The photoresponse exhibited a three order of magnitude increase in the short-circuit current, (0.5±0.2) nA, with respect to the dark current (Supplementary Fig. 5) and an open-circuit voltage of (270±120) mV. It is worth mentioning that average short-circuit current and open-circuit voltage for the single component **1** were measured as (0.09±0.01) nA and (160±60) mV, respectively. The reported open-circuit voltage values are close to the energy level difference between the highest occupied molecular orbital (HOMO) of **1** and the work function of graphene. The electrode work function is 4.5 eV (ref. 54) for graphene and 4.3 eV (ref. 55) for gallium. The calculated first excitation of **1** occurs at 1.6 eV (775 nm) and the HOMO of **1** is 4.8 eV below the vacuum level (Supplementary Fig. 6). The correlation with the energy level difference might be coincidental, as the origins of the open-circuit voltage in excitonic solar cells are under intense discussion^56^. In our setup, a full molecular monolayer is guaranteed by employing concentrations and volumes, equivalent to 3.8 × 10^13^ molecules of **1** per substrate (and similar amount for **2**). Considering a substrate area of 5 × 5 mm^2^ and three TDI molecules per **1+2** unit cell area of 13.8 nm^2^, a monolayer is formed with 5.4 × 10^12^ TDI molecules. The higher amount of molecules applied to the substrate was employed to compensate for ring stain effects when drying drop-casting solutions (see Supplementary Fig. 1). A peak UV–vis absorbance of 0.012 at (735±2) nm for **1** provides additional evidence of a molecular monolayer. Upon **1+2** supramolecular network formation, absorbance is reduced, as expected because the assembly of a porous architecture entails a lower molecular surface density. In addition, formation of *π*-aggregate layers^49^,^50^, reduces the resulting absorbance cross section, as well as hydrogen bonding^57^ where charge transfer is likely to occur^58^.

Our estimate on the tunnelling contact area allows us to elaborate on the technological implications of photoresponsive surface assemblies. Previously reported optimized photovoltaic devices of thin films of precursor molecule **7** blended with an organic acceptor^59^, featured IPCEs^35^ of 0.3% at 700 nm. These thin films were prepared by spin-coating solutions of 13 mg ml^−1^. The comparable estimate of an IPCE of (0.6±0.25)% at 710 nm for our system prepared by drop-casting a 5 μl of a solution 1,000 times more diluted (0.015 mg ml^−1^ or approximately 12 μM) suggests that the photovoltaic response of few monolayers of self-assembled molecular architectures could outperform the response of bulk spin-coated materials. This is in part due to high internal quantum yields for monolayer absorbers or high collection efficiencies at interfaces, as reported for C_60_-porphyrin mixed self-assembled dyads^60^, natural photosystem-I^18^,^61^ and naturally occurring 2D crystals^62^,^63^. In our configuration, two molecular interfaces are formed, one between **1+2** on GHD and one between **1+2** and gallium oxide on gallium. The oxide^64^ tunnelling barrier between gallium and the supramolecular assembly forms a blocking-layer that prevents the efficient collection of photogenerated electrons at the gallium electrode. We suggest that the resulting photoresponsive device element is hole-only, that is, photogenerated holes are readily collected at the graphene photoanode, while electrons have to tunnel to the gallium electrode. Hence, it is expected that the tuning of the work function and of the appropriate tunnelling junction material (for example, allowing hole-only and electron-only transport in the pertinent contacts)^65^ will radically improve the efficiency of monolayer-thin organic devices.

In summary, bottom-up modular self-assembled networks and 2D materials grant access to device fabrication with molecular precision. We have shown the first macroscopic (μm scale) photoresponse characterization of a bicomponent supramolecular interfacial assembly. By *ex situ* estimations of the tunnelling area, our non-optimized device element configuration yields an IPCE (at 710 nm) as high as 0.6% in air, opening novel avenues towards tandem photovoltaics from monolayer-thin sensitizers. More importantly, we highlighted how, more than a decade since the introduction of interfacial bottom-up modular self-assembly^21^,^23^,^24^ and *in situ* on-surface synthesis^25^ for atomically precise fabrication, serious efforts are still required for molecularly precise device fabrication, with high throughput, large-area, dedicated analytical methods simply lacking. Our work motivates rapid progress in molecular engineered manufacturing and monolayer-by-monolayer molecular printing methods, which potentially grant access to exponential optimization of device performance.

## Methods

### CVD-grown graphene transfer and network formation

The experiments were performed under ambient conditions on CVD-grown graphene (10 × 10 mm on a copper foil, Graphene Platform, Japan) on hydrogenated diamond (Element Six, thickness: 500 μm) surface. A high purity diamond C-(100) plate was cleaned with pure *N*-methyl-2-pyrrolidone (NMP) and isopropanol sonication treatment. Oxidation of the substrate was conducted with oxygen microwave plasma (TePla 100-E): 600 s at 200 W load coil power and 50 Pa oxygen pressure at a constant oxygen flow rate equivalent to 90 cm^3^ min^−1^ (SCCM). To ensure a high quality conductive termination, hydrogenation of the diamond surface was performed in a quartz tube reactor (Seki Technotron Corp.) of a microwave-coupled ASTEX plasma system with three steps: 750 W and 50 mbar hydrogen pressure at a constant hydrogen flow rate of 100 SCCM at 700 °C for 15 min; 230 W, 10 mbar hydrogen pressure, 100 SCCM at 300 °C for 10 min; 0 W, 10 mbar hydrogen pressure, 100 SCCM at 35 °C for 30 min. The hydrogenated diamond was characterized via STM (Supplementary Fig. 7). After these treatments, the graphene layer was transferred^66^,^67^ on diamond H-C(100). Melamine (Fluka, 52549, 99%) was used as received. Dimethyl sulfoxide (DMSO, Sigma-Aldrich, 99.9%) and anhydrous 1,2,4-trichlorobenzene (TCB, Sigma-Aldrich, 99.9%) were used as solvents without further purification. Uncertainty values were derived from the standard deviation of the balance's linearity (Sartorius CPA2245). The mother solutions in 10% DMSO and 90% in TCB were sonicated and heated to 80 °C and a dilution was prepared in TCB with a concentration ratio of **1+2** of 12 μM:8 μM. The network was obtained by drop-casting (5±1) μl of **1+2** on GHD.

### Scanning tunnelling microscopy measurements

STM measurements (Agilent Technologies 5,100) were performed in constant current and constant height mode. The scanning tips were prepared by mechanically cutting a Pt/Ir wire (80:20%, Goodfellow, UK). STM data were analysed with the free WSxM software (Nanotec Electronica S.L., Spain) and the Gwyddion software^68^. All images, except Fig. 3c with Gaussian filtering, are shown with line-wise flattening to remove tilting effect of the substrate plane. The network structures were modelled by the Merck modular force field (MMFF)^69^. Supplementary Fig. 8 reports the STM data of the molecule of **1** in HOPG.

### UV–visible absorption spectroscopy

UV–vis measurements were performed with a UV/VIS/NIR Spectrometer, Lambda 900 (Perkin Elmer). The spectra were recorded at the full spectra range (2,000–200 nm) with 5 nm data interval, 0.32 and 0.68 s integration time for UV–vis and NIR, respectively. For absorption measurements, (5±1) μl of **1+2** (12 μM:8 μM) in 1,2,4-Trichlorobenzene (TCB, Sigma-Aldrich, 99.9%) were drop-casted on GHD and dried in air. The spectrum was averaged 10 times and is plotted versus the wavelength for each sample.

### Photoresponse measurements

*IV* measurements were performed with a home-built STM described in detail elsewhere^70^. A blunt tungsten tip was dipped into a heated gallium droplet and directly mounted into the tip holder of the scanner. The gallium electrode was approached to the surface with approach parameters of 2 nA and 100 mV. The spectroscopy data were recorded with a forward sweep rate of 200 mV s^−1^ using a Femto pre-amplifier. For recording the on–off cycle photovoltages in Fig. 5g, EGaIn electrodes coated with alkyl thiols were employed for increased stability. The tungsten blunt tips were dipped in EGaIn (495425, Sigma-Aldrich) until a smooth coating was obtained and subsequently immersed in a pure solution of 1-dodecanethiol (471364, Sigma-Aldrich) for 15 min. These *IV* measurements were independently performed in an Agilent Technologies 5,100 using a logarithmic current amplifier to avoid current saturation. Before each single tunnelling spectroscopy measurement, the feedback vertical position of the electrode was regulated again to a tunnelling current of 1 nA and a voltage of 300 mV and turned off. The data were recorded with a forward sweep rate of 20 mV s^−1^. For the illumination, a 710 nm (30 mW, 18°, Roithner Lasertechnik, Austria) and 520 nm (9,600 mCd, 123 mW, 30°, Nichia, Japan) LED were used. The photovoltaic detection limit of our setup is 90 mV, calculated as trice the standard deviation of the dark voltage. The acquired data in Fig. 5c,d,g corresponds to stable contact junctions (no observable oscillations in the junction *z* axis piezo electric control nor identifiable non-contact tunnelling junction formation, see main text) among tenths of different area surveys on four different samples.

### Fluorescence measurement of the Ga droplet contact area

A Ga droplet was prepared as employed for the photocurrent measurements. The droplet was brought into tunnelling contact with a thin film of fluorescent dye on HOPG surface with the same approach and current parameters used in the STM measurement. By employing a nm-thick film of an insulating dye Rhodamin B (Radiant Dyes, Wermelskirchen), a contact junction forms, implying physical contact with the monolayer. The physical contact of the gallium droplet with a thick film of Rhodamin B leads to a higher deformation the droplet, increasing the contact area in comparison to the photoresponse contacts. Therefore, this method accurately estimates an upper bound to the actual device element contact area and therefore, a minimum current density and efficiency. To form the nm-thick film, HOPG was spin-coated (480 r.p.m.) three times with 10 μl of a saturated solution of Rhodamin B in acetone, and dried between each application. The tip was retracted from the surface, fixed to a microscope slide and imaged under fluorescence conditions with a fluorescence microscope (Leica DMI 3000B, Wetzlar). The image of the tip is shown in Supplementary Fig. 2 with enhanced contrast to make the contour of the tip visible (darker area). From the raw data (greyscale TIFF-image), the number of pixels with brightness higher than a certain threshold was extracted. From the length scale per pixel (known from calibration), the total area corresponding to the pixels was calculated. The threshold was set to a clear gap between brighter and darker pixels in the histogram and the corresponding area in the image was identified.

## Additional information

**How to cite this article:** Wieghold, S. *et al*. Photoresponse of supramolecular self-assembled networks on graphene-diamond interfaces. *Nat. Commun.* 7:10700 doi: 10.1038/ncomms10700 (2016).

## Supplementary Material

Supplementary Information Supplementary Figures 1-8, Supplementary Methods and Supplementary References.

## Figures and Tables

**Figure 1:**
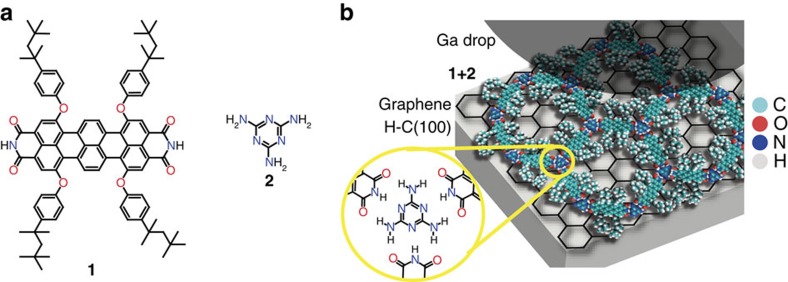
Chemical structures of the molecules and setup. (**a**) Structure of TDI tetracarboxylic acid derivative (**1**) and melamine (**2**) (**b**). Schematic drawing of the photoresponse device setup including the ideal representation of the **1+2** mixture yielding a [**1**_3_**2**_2_]_n_ hexagonal supramolecular network via hydrogen bonds (yellow circle).

## References

[b1] Tang C. W. & VanSlyke S. A. Organic electroluminescent diodes. Appl. Phys. Lett. 51, 913–915 (1987).

[b2] Tang C. W. 2-Layer organic photovoltaic cell. Appl. Phys. Lett. 48, 183–185 (1986).

[b3] Burroughes J. H. . Light-emitting diodes based on conjugated polymers. Nature 347, 539–541 (1990).

[b4] Hebner T. R., Wu C. C., Marcy D., Lu M. H. & Sturm J. C. Ink-jet printing of doped polymers for organic light emitting devices. Appl. Phys. Lett. 72, 519–521 (1998).

[b5] de Boer R. W. I., Gershenson M. E. & Morpurgo A. F. Podzorov V., Organic single-crystal field-effect transistors. Phys. Status Solidi A 201, 1302–1331 (2004).

[b6] Halik M. & Hirsch A. The potential of molecular self-assembled monolayers in organic electronic devices. Adv. Mater. 23, 2689–2695 (2011). 21823250 10.1002/adma.201100337

[b7] Yamamoto Y. . Photoconductive coaxial nanotubes of molecularly connected electron donor and acceptor layers. Science 314, 1761–1764 (2006). 17170300 10.1126/science.1134441

[b8] Wasielewski M. R. Self-assembly strategies for integrating light harvesting and charge separation in artificial photosynthetic systems. Acc. Chem. Res. 42, 1910–1921 (2009). 19803479 10.1021/ar9001735

[b9] Briseno A. L. . Patterning organic single-crystal transistor arrays. Nature 444, 913–917 (2006). 17167482 10.1038/nature05427

[b10] Murphy A. R. & Fréchet J. M. J. Organic semiconducting oligomers for use in thin film transistors. Chem. Rev. 107, 1066–1096 (2007). 17428023 10.1021/cr0501386

[b11] Heeger A. J. 25th anniversary article: bulk heterojunction solar cells: understanding the mechanism of operation. Adv. Mater. 26, 10–27 (2014). 24311015 10.1002/adma.201304373

[b12] Vlachopoulos N., Liska P., Augustynski J. & Grätzel M. Very efficient visible-light energy harvesting and conversion by spectral sensitization of high surface-area polycrystalline titanium-dioxide films. J. Am. Chem. Soc. 110, 1216–1220 (1988).

[b13] O'Regan B. & Grätzel M. A low-cost, high-efficiency solar-cell based on dye-sensitized colloidal TiO_2_ films. Nature 353, 737–740 (1991).

[b14] Hagfeldt A., Boschloo G., Sun L. C., Kloo L. & Pettersson H. Dye-sensitized solar cells. Chem. Rev. 110, 6595–6663 (2010). 20831177 10.1021/cr900356p

[b15] Scharber M. C. . Design rules for donors in bulk-heterojunction solar cells - towards 10% energy-conversion efficiency. Adv. Mater. 18, 789–794 (2006).

[b16] Krebs F. C., Espinosa N., Hosel M., Sondergaard R. R. & Jorgensen M. 25th anniversary article: rise to power-OPV-based solar parks. Adv. Mater. 26, 29–38 (2014). 24741693 10.1002/adma.201302031

[b17] Park H. . Nanomechanical oscillations in a single-C-60 transistor. Nature 407, 57–60 (2000). 10993069 10.1038/35024031

[b18] Gerster D. . Photocurrent of a single photosynthetic protein. Nat. Nanotechnol. 7, 673–676 (2012). 23023644 10.1038/nnano.2012.165

[b19] Palma C.-A. & Samorì P. Blueprinting macromolecular electronics. Nat. Chem. 3, 431–436 (2011). 21602856 10.1038/nchem.1043

[b20] Rabe J. P. & Buchholz S. Commensurability and mobility in two-dimensional molecular patterns on graphite. Science 253, 424–427 (1991). 17746397 10.1126/science.253.5018.424

[b21] Theobald J. A., Oxtoby N. S., Phillips M. A., Champness N. R. & Beton P. H. Controlling molecular deposition and layer structure with supramolecular surface assemblies. Nature 424, 1029–1031 (2003). 12944962 10.1038/nature01915

[b22] Palma C.-A. . Tailoring bicomponent supramolecular nanoporous networks: phase segregation, polymorphism, and glasses at the solid-liquid interface. J. Am. Chem. Soc. 131, 13062–13071 (2009). 19702301 10.1021/ja9032428

[b23] Stepanow S. . Steering molecular organization and host-guest interactions using two-dimensional nanoporous coordination systems. Nat. Mater. 3, 229–233 (2004). 15004551 10.1038/nmat1088

[b24] Barth J. V. Molecular architectonic on metal surfaces. Annu. Rev. Phys. Chem 58, 375–407 (2007). 17430091 10.1146/annurev.physchem.56.092503.141259

[b25] Grill L. . Nano-architectures by covalent assembly of molecular building blocks. Nat. Nanotechnol. 2, 687–691 (2007). 18654406 10.1038/nnano.2007.346

[b26] Zwaneveld N. A. . Organized formation of 2D extended covalent organic frameworks at surfaces. J. Am. Chem. Soc. 130, 6678–6679 (2008). 18444643 10.1021/ja800906f

[b27] Ciesielski A. . Dynamic covalent chemistry of bisimines at the solid/liquid interface monitored by scanning tunnelling microscopy. Nat. Chem. 6, 1017–1023 (2014). 25343608 10.1038/nchem.2057

[b28] Barth J. V., Costantini G. & Kern K. Engineering atomic and molecular nanostructures at surfaces. Nature 437, 671–679 (2005). 16193042 10.1038/nature04166

[b29] Ciesielski A., Palma C.-A., Bonini M. & Samorì P. Towards supramolecular engineering of functional nanomaterials: pre-programming multi-component 2D self-assembly at solid-liquid interfaces. Adv. Mater. 22, 3506–3520 (2010). 20626011 10.1002/adma.201001582

[b30] Palma C.-A., Cecchini M. & Samorì P. Predicting self-assembly. Chem. Soc. Rev. 41, 3713–3730 (2012). 22430648 10.1039/c2cs15302e

[b31] Whitelam S. . Common physical framework explains phase behavior and dynamics of atomic, molecular, and polymeric network formers. Phys. Rev. X 4, 011044–1 011044-12 (2014).

[b32] Blunt M. O. . Guest-induced growth of a surface-based supramolecular bilayer. Nat. Chem. 3, 74–78 (2011). 21160521 10.1038/nchem.901

[b33] Li J. . Three-dimensional bicomponent supramolecular nanoporous self-assembly on a hybrid all-carbon atomically flat and transparent platform. Nano Lett. 14, 4486–4492 (2014). 25115337 10.1021/nl501452s

[b34] Halls J. J. M. . Efficient photodiodes from interpenetrating polymer networks. Nature 376, 498–500 (1995).

[b35] Gunes S., Neugebauer H. & Sariciftci N. S. Conjugated polymer-based organic solar cells. Chem. Rev. 107, 1324–1338 (2007). 17428026 10.1021/cr050149z

[b36] Zerkowski J. A., Seto C. T. & Whitesides G. M. Solid-state structures of rosette and crinkled tape motifs derived from the cyanuric acid melamine lattice. J. Am. Chem. Soc. 114, 5473–5475 (1992).

[b37] Palma C.-A., Samorì P. & Cecchini M. Atomistic simulations of 2D bicomponent self-assembly: from molecular recognition to self-healing. J. Am. Chem. Soc. 132, 17880–17885 (2010). 21114285 10.1021/ja107882e

[b38] Weil T., Vosch T., Hofkens J., Peneva K. & Müllen K. The rylene colorant family--tailored nanoemitters for photonics research and applications. Angew. Chem. Int. Ed. Engl. 49, 9068–9093 (2010). 20973116 10.1002/anie.200902532

[b39] Wöll D. . Polymers and single molecule fluorescence spectroscopy, what can we learn? Chem. Soc. Rev. 38, 313–328 (2009). 19169450 10.1039/b704319h

[b40] Chen L., Li C. & Müllen K. Beyond perylene diimides: synthesis, assembly and function of higher rylene chromophores. J. Mater. Chem. C 2, 1938–1956 (2014).

[b41] Li G., Zhu R. & Yang Y. Polymer solar cells. Nat. Photon. 6, 153–161 (2012).

[b42] Nolde F. . Synthesis and modification of terrylenediimides as high-performance fluorescent dyes. Chem. Eur. J. 11, 3959–3967 (2005). 15844133 10.1002/chem.200401177

[b43] Avlasevich Y., Li C. & Müllen K. Synthesis and applications of core-enlarged perylene dyes. J. Mater. Chem. 20, 3814–3826 (2010).

[b44] Würthner F., Bay-Substituted Perylene & Bisimides, Twisted fluorophores for supramolecular chemistry. Pure Appl. Chem. 78, 2341–2349 (2006).

[b45] Madueno R., Raisanen M. T., Silien C. & Buck M. Functionalizing hydrogen-bonded surface networks with self-assembled monolayers. Nature 454, 618–621 (2008). 18668104 10.1038/nature07096

[b46] Huang S. . Molecular selectivity of graphene-enhanced Raman scattering. Nano Lett. 15, 2892–2901 (2015). 25821897 10.1021/nl5045988

[b47] Würthner F. . Preparation and characterization of regioisomerically pure 1,7-disubstituted perylene bisimide dyes. J. Org. Chem. 69, 7933–7939 (2004). 15527273 10.1021/jo048880d

[b48] Pschirer N. G., Kohl C., Nolde T., Qu J. Q. & Müllen K. Pentarylene- and hexarylenebis(dicarboximide)s: near-infrared-absorbing polyaromatic dyes. Angew. Chem. Int. Ed. Engl. 45, 1401–1404 (2006). 16425337 10.1002/anie.200502998

[b49] Chen Z. J. . Photoluminescence and conductivity of self-assembled π-π stacks of perylene bisimide dyes. Chem. Eur. J. 13, 436–449 (2007). 17143925 10.1002/chem.200600889

[b50] Spano F. C. The spectral signatures of frenkel polarons in h- and j-aggregates. Acc. Chem. Res. 43, 429–439 (2010). 20014774 10.1021/ar900233v

[b51] Weigand R., Rotermund F. & Penzkofer A. Aggregation dependent absorption reduction of indocyanine green. J. Phys. Chem. A 101, 7729–7734 (1997).

[b52] Nijhuis C. A., Reus W. F., Siegel A. C. & Whitesides G. M. A molecular half-wave rectifier. J. Am. Chem. Soc. 133, 15397–15411 (2011). 21842878 10.1021/ja201223n

[b53] Nijhuis C. A., Reus W. F., Barber J. R. & Whitesides G. M. Comparison of SAM-based junctions with Ga_2_O_3_/Egaln top electrodes to other large-area tunnelling junctions. J. Phys. Chem. C 116, 14139–14150 (2012).

[b54] Giovannetti G. . Doping graphene with metal contacts. Phys. Rev. Lett. 101, 026803–1 026803-4 (2008). 18764212 10.1103/PhysRevLett.101.026803

[b55] Srisonphan S., Jung Y. S. & Kim H. K. Metal-oxide-semiconductor field-effect transistor with a vacuum channel. Nat. Nanotechnol. 7, 504–508 (2012). 22751220 10.1038/nnano.2012.107

[b56] Gregg B. A. Excitonic solar cells. J. Phys. Chem. B. 107, 4688–4698 (2003).

[b57] Llanes-Pallas A. . Engineering of supramolecular H-bonded nanopolygons via self-assembly of programmed molecular modules. J. Am. Chem. Soc. 131, 509–520 (2009). 19105700 10.1021/ja807530m

[b58] Reece S. Y. & Nocera D. G. Proton-coupled electron transfer in biology: results from synergistic studies in natural and model systems. Annu. Rev. Biochem. 78, 673–699 (2009). 19344235 10.1146/annurev.biochem.78.080207.092132 PMC4625787

[b59] Gorenflot J. . Detailed study of N,N '-(diisopropylphenyl)-terrylene-3,4:11,12-bis(dicarboximide) as electron acceptor for solar cells application. Synth. Met. 161, 2669–2676 (2012).

[b60] Imahori H. & Fukuzumi S. Porphyrin- and fullerene-based molecular photovoltaic devices. Adv. Funct. Mater. 14, 525–536 (2004).

[b61] Carmeli I., Frolov L., Carmeli C. & Richter S. Photovoltaic activity of photosystem I-based self-assembled monolayer. J. Am. Chem. Soc. 129, 12352–12353 (2007). 17880215 10.1021/ja073040c

[b62] Bernardi M., Palummo M. & Grossman J. C. Extraordinary sunlight absorption and one nanometer thick photovoltaics using two-dimensional monolayer materials. Nano Lett. 13, 3664–3670 (2013). 23750910 10.1021/nl401544y

[b63] Lee C. H. . Atomically thin P-N junctions with van Der Waals heterointerfaces. Nat. Nanotechnol. 9, 676–681 (2014). 25108809 10.1038/nnano.2014.150

[b64] Čechal J. . Characterization of oxidized gallium droplets on silicon surface: an ellipsoidal droplet shape model for angle resolved X-ray photoelectron spectroscopy analysis. Thin Solid Films 517, 1928–1934 (2009).

[b65] Shieh J. T. . The effect of carrier mobility in organic solar cells. J. Appl. Phys. 107, 084503–1 084503-9 (2010).

[b66] Yan Z. . Toward the synthesis of wafer-scale single-crystal graphene on copper foils. ACS Nano 6, 9110–9117 (2012). 22966902 10.1021/nn303352k

[b67] Li X. . Large-area synthesis of high-quality and uniform graphene films on copper foils. Science 324, 1312–1314 (2009). 19423775 10.1126/science.1171245

[b68] Nečas D. & Klapetek P. Gwyddion: an open-source software for SPM data analysis. Cent. Eur. J. Phys. 10, 181–188 (2012).

[b69] Halgren T. A. Merck molecular force field. I. Basis, form, scope, parameterization, and performance of MMFF94. J. Comput. Chem. 17, 490–519 (1996).

[b70] Wilms M., Kruft M., Bermes G. & Wandelt K. A new and sophisticated electrochemical scanning tunneling microscope design for the investigation of potentiodynamic processes. Rev. Sci. Instrum. 70, 3641–3650 (1999).

