# Supplementary material for: Photoresponse of supramolecular self-assembled networks on graphene–diamond interfaces
Source: Nat Commun. 2016 Feb 25;7:10700. doi: 10.1038/ncomms10700 (PMC4773422; doi:10.1038/ncomms10700)
Supplement: Supplementary Information — Supplementary Figures 1-8, Supplementary Methods and Supplementary References. [file ncomms10700-s1.pdf]

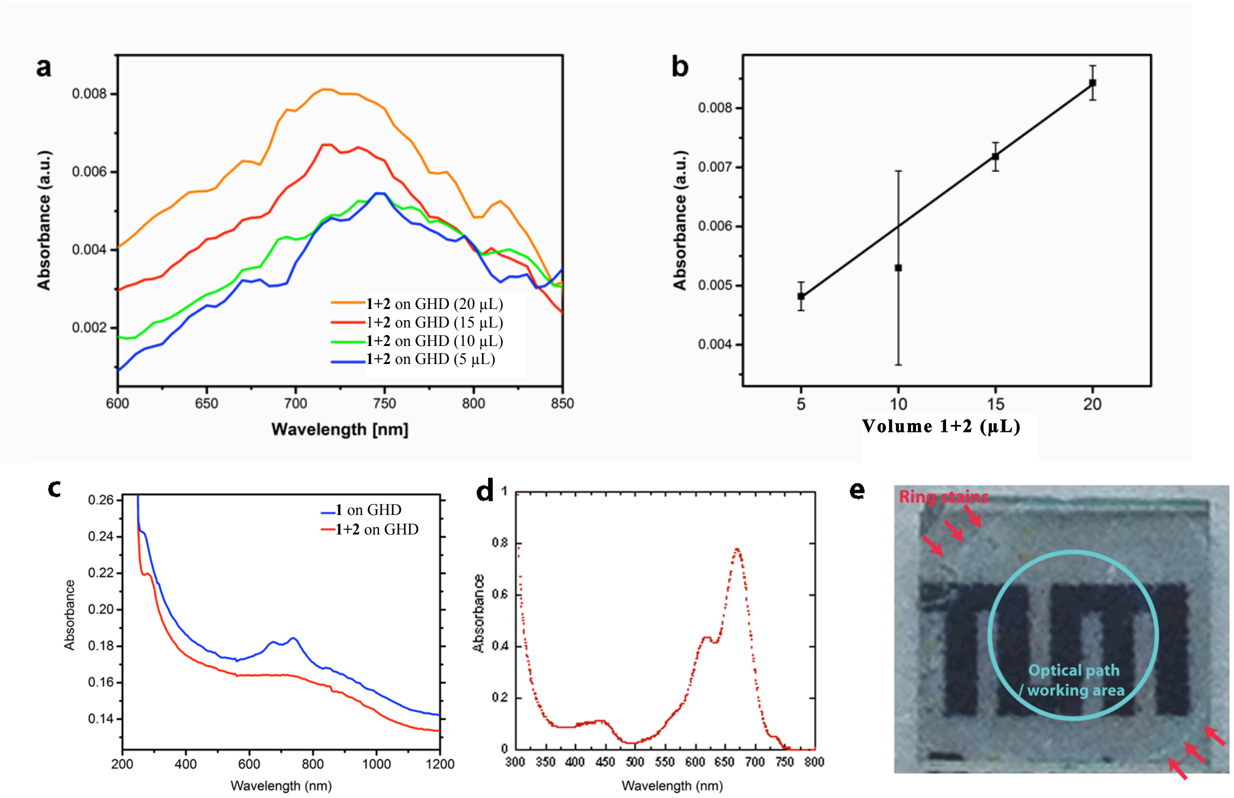

Supplementary Figure 1 | UV-Vis measurements a. UV-Vis spectroscopy of drop-casted volume of 1+2 (24 µM:16 µM) on GHD for 5 µL, 10 µL, 15 µL and 20 µL in arbitrary units. **b.** Absorbance in arbitrary units at 710 nm with error bars (10 averages of spectra) as a function of the volume dropped onto the surface. UV-Vis measurements were performed by stepwise adding 5±1 µL of **1** and **2** (24 µM:16 µM) and letting the films dry. A linear increase in absorbance at 710 nm was detected, but even after five drop-casted equivalents did not reach 0.01 absorbance units (the peak-to-baseline absorbance of 5±1 µL of 12µ M pristine **1**). Error bars are s.d. for three different samples. **c.** Absolute absorbance of **1+2** (12 µM:8 µM) and **1** (12 µM). Note that the overall higher absolute absorbance of **1** vs. **1+2** observed in over several freshly prepared samples can be potentially assigned to a decrease in the reflectivity of the atomically flat diamond crystal with 1+2 and does not necessarily signifies a lower absolute absorption of the GHD substrate with the 1+2 active layer. **d.** UV-Vis measurement of **1** in quartz cuvettes in 1% DMSO in TCB solutions, λ_{max} = 669 nm. **e.** Image of a GHD sample preparation after overnight evaporation of 5±1 µL of 12µM:8µM of 1+2. Ring staining is observed at the border of the sample. The studied area is circled.

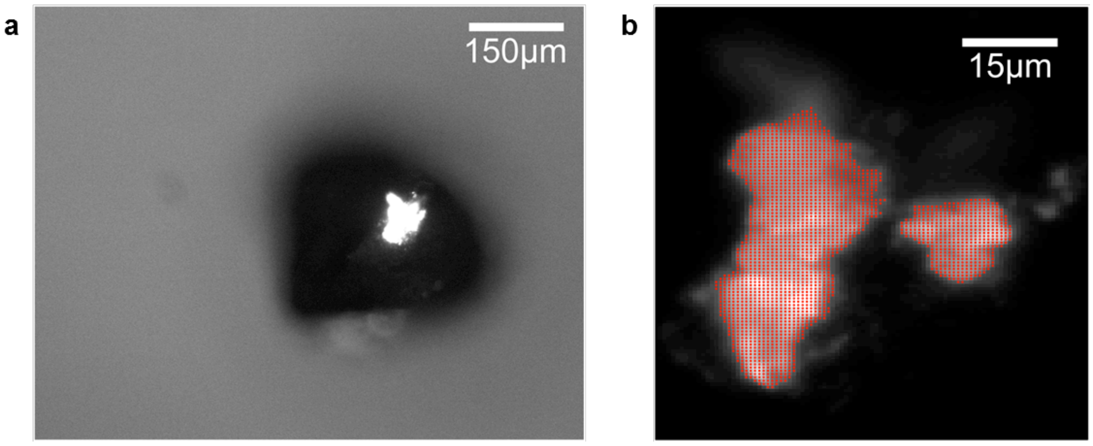

Supplementary Figure 2 | Ex-situ fluorescence measurements estimating the tunneling contact area of the Ga droplet on the 1+2 assemblies. A film of Rhodamine B was spin coated on HOPG surface, brought into tunneling contact with the Ga droplet electrode and the fluorescence image was recorded. **a.** Fluorescence image of the tip with enhanced contrast. **b.** Threshold image used to determine the contact area (red).

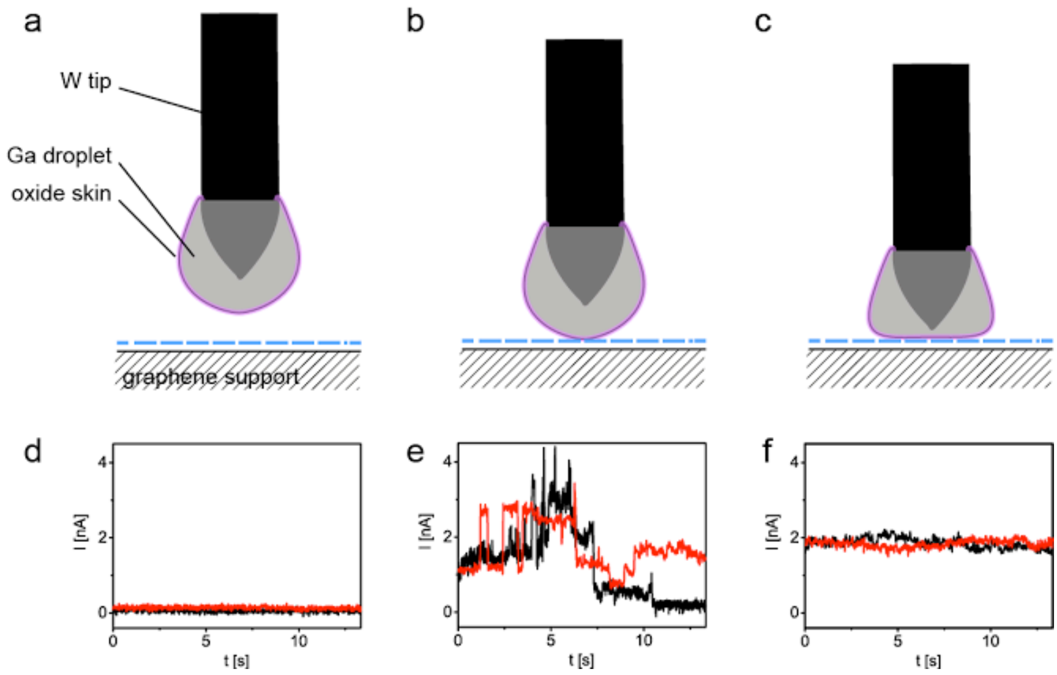

Supplementary Figure 3 | Current characteristics of the junction after automatic Ga droplet approach a,d. Current with bthe tip far away from the substrate. **b,e.** Junction while approaching, indicating that intermittent contact with the sample occurs, possibly during deformation. **c,f.** Stable junction (contact regime). Approach setpoint 2 nA, sample voltage 100 mV, approach velocity 8 nm s^{-1}.

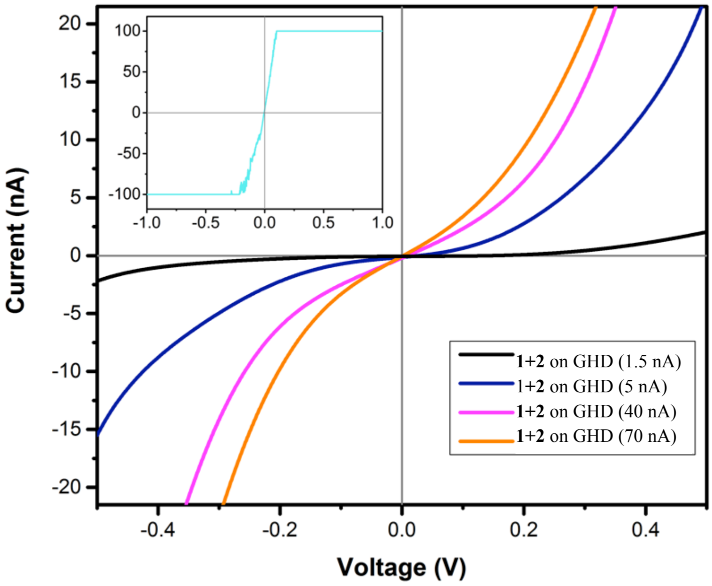

Supplementary Figure 4 | Dark tunneling spectra for different approach setpoint parameters employing biases of 100 mV. Inset: Spectra with an approach parameter of 100 nA.

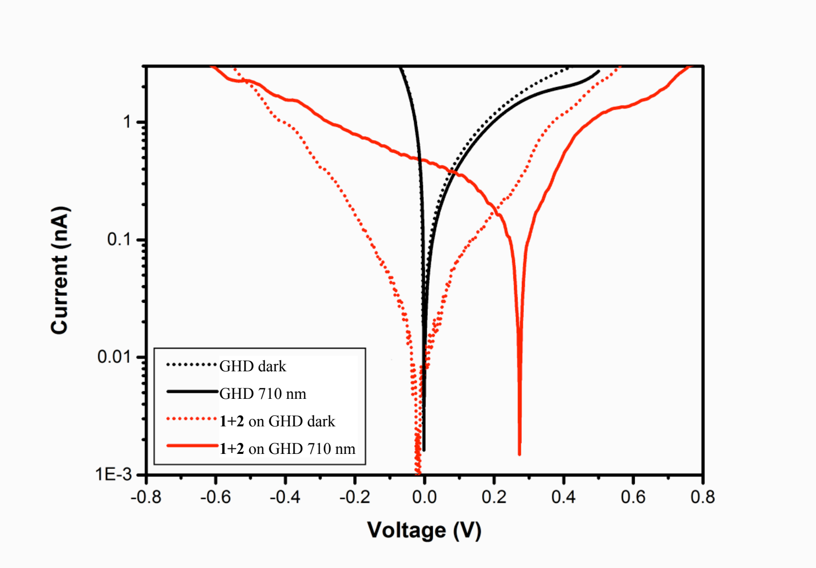

Supplementary Figure 5 | IV characteristics in a semi-log plot of Fig. 5c in the main text.

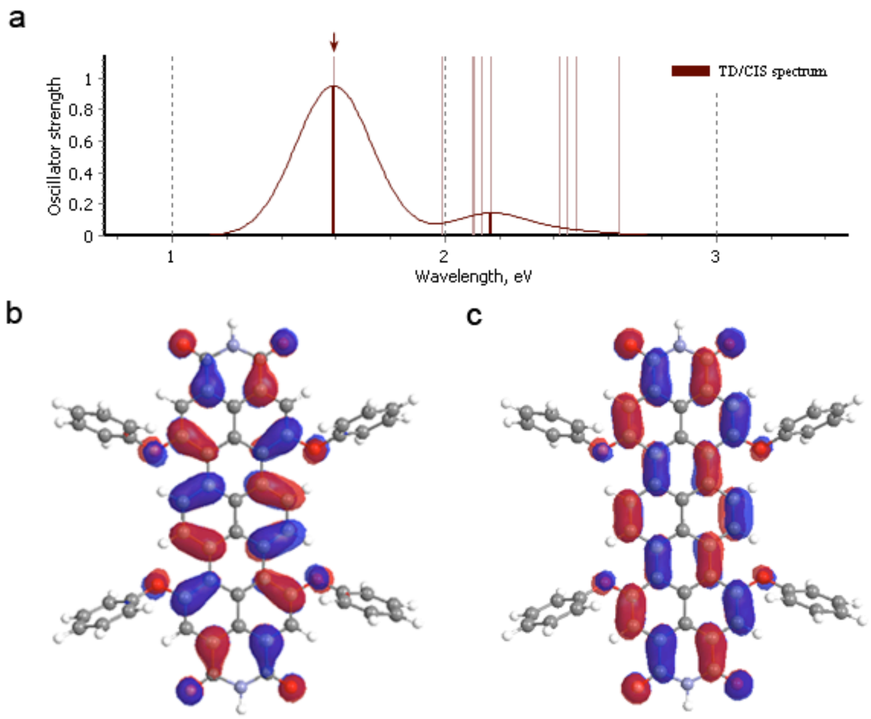

Supplementary Figure 6 | Time-dependent density functional theory of 1 a. Excitation spectrum for 1 calculated at the PBE 96 [1] level of theory with a 6-311G basis set employing the first 10 excited states. The first excitation at 1.6 eV arises mainly from the HOMO-LUMO transition. Molecular orbital rendering of **b.** the HOMO and **c.** the LUMO of the molecule. TD-DFT calculations were performed with the Firefly package (Granovsky AA. Firefly Version 8.0, www http://classic.chem.msu.su/gran/firefly/index.html. 2014) and structures optimized to an RMS energy gradient below 0.00001 Hartree.

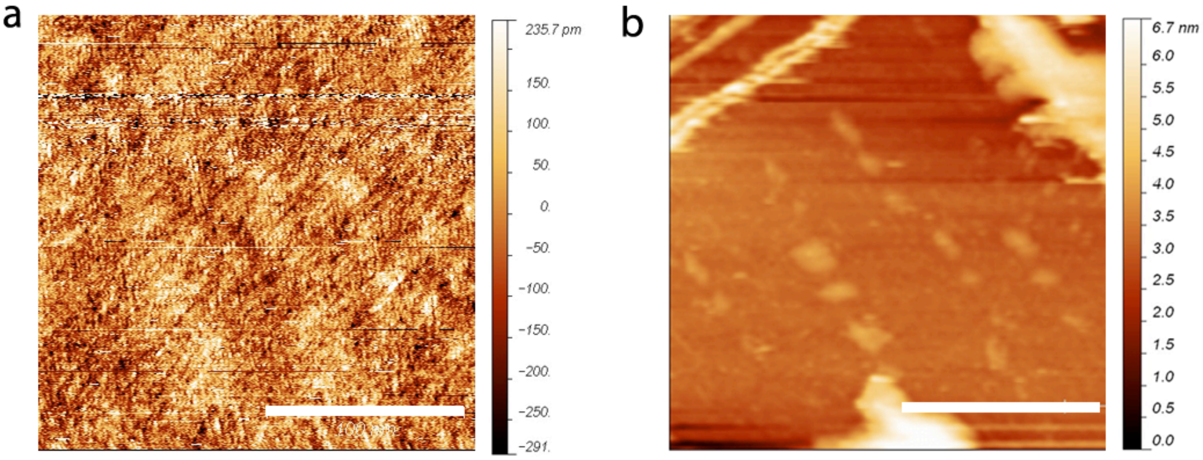

Supplementary Figure 7 | Large area STM topography of atomically flat H-C(100) diamond surface a. before and **b.** after CVD graphene transfer. Images rendered by the Gwyddion program [2]. Scale bar 100 nm.

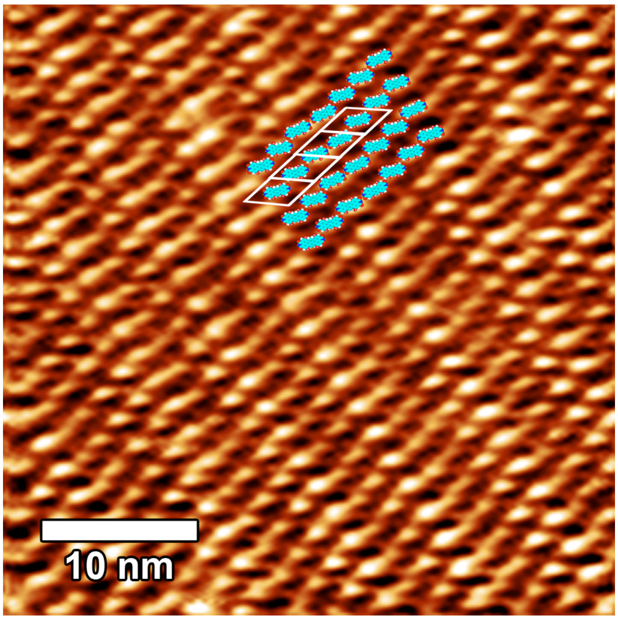

Supplementary Figure 8 | STM image of 1b 12 μM monolayer of **1** on HOPG (in TCB solvent with 1-2% DMSO). Unit cell a=2.5 nm b=2.3 nm, (a,b)=45°. Area 4.0 nm^2. Tentative molecular models of **1** in the image area shown without alkyl side chains.

Supplementary Methods

1,6,9,14-tetrakis(4-*tert*octylphenoxy)terrylene-3,4:11,12-tetracarboxdianhydride 8.

N,N'-bis(2,6-di*iso*propylphenyl)-1,6,9,14-tetrakis(4-*tert*octylphenoxy)terrylene-3,4:11,12-diimide obtained as indicated in supplementary ref. 3 (165 mg, 0.1 mmol), KOH (0.5 g), KF (0.5 g) and 2-methyl-2-butanol (50 mL) were placed into 100 ml round-bottom flask and refluxed for 3 h. Reaction mixture was cooled to room temperature and the solvent was evaporated under vacuum to dryness. The residue was dissolved in concentrated acetic acid (30 mL) and stirred for 1 h at room temperature, then diluted with water (50 mL). The precipitate was filtered, rinsed with water (100 mL), methanol (50 mL) and purified by column chromatography on silica gel using toluene as an eluent. The first fraction was collected to give N-(2,6-di*iso*propylphenyl)-1,6,9,14-tetrakis(4-*tert*octylphenoxy) terrylene-3,4-dicarboximide-11,12-dicarboxanhydride 33 mg (22%).

The second fraction was collected to give the title compound **8**, 70 mg (53%). **^{1}H-NMR (250 MHz, $C_2D_2Cl_4$, 25 °C):** δ= 9.49 (s, 4H), 8.04 (s, 4H), 7.39 (d, ^3J=7.4 Hz, 8H), 7.04 (d, ^3J=7.0 Hz, 8H), 1.67 (s, 8H), 1.34 (s, 24H), 0.70 ppm (s, 36H); **FD-MS** (m/z, rel.int.) 1332.3 (100%) [M^+].

1,6,9,14-tetrakis(4-*tert*octylphenoxy)terrylene-3,4:11,12-diimide 1.

1,6,9,14-tetrakis(4-*tert*octylphenoxy)terrylene-3,4:11,12-tetracarboxdianhydride **8** (50 mg,0.0375 mmol) and ammonium acetate (1 g) were suspended in propionic acid (20 ml) and stirred under reflux for 20 h. The reaction mixture was cooled to room temperature, diluted with water (30 ml) and filtered. The precipitate was rinsed with water (100 ml), methanol (50 ml) and dried under vacuum. The solid was purified by column chromatography on silica gel (dichloromethane/THF, 50:1) to give the title compound 10 mg (20%). **^{1}H-NMR (250 MHz, $C_2D_2Cl_4$, 25 °C):** δ= 9.44 (s, 4H), 8.33 (s, 2H), 8.07 (s, 4H), 7.36 (d, ^3J=8.8 Hz, 8H), 7.02 (d, ^3J=8.6 Hz, 8H), 1.65 (s, 8H), 1.32 (s, 24H), 0.68 ppm (s, 36H); UV-Vis ($C_6H_3Cl_3/C_2H_6SO$, 99:1): λ_{max} = 669 nm; **FD-MS** (m/z, rel.int.): [M^+] calcd. for $C_{90}H_{93}N_2O_8$ 1330.7; found 1330.4 (100%), ^{13}C-NMR data could not be recorded due to the low solubility of the product. STM data in the main text complements structural data.

The incident photon-to-current efficiency (*IPCE*) at the irradiation wavelength of 710 nm, or external quantum efficiency (*EQE*) is defined as equation (1):

$$\text{IPCE(710nm)}=\frac{\text{generated electron flux density}}{\text{incident photon flux density}}=\frac{\rho_e[\text{m}^{-2}\text{s}^{-1}]}{\rho_{ph,i}[\text{m}^{-2}\text{s}^{-1}]}=(0.62\pm0.25)\% \qquad (1)$$

The generated electron flux density can be calculated following equation (2):

$$\rho_{\mathrm{e}}[\mathrm{m^{-2}s^{-1}}] = \frac{I_{\mathrm{t}}}{A_{\mathrm{cont}} \cdot e} = (3.0 \pm 1.1) \cdot 10^{18}\,\mathrm{m^{-2}s^{-1}} \qquad (2)$$

The incident photon flux density was calculated according to equation (3):

$$\rho_{\mathrm{ph,i}}[\mathrm{m^{-2}s^{-1}}] = \frac{P_{\mathrm{LED}} \cdot T}{E_{\mathrm{ph}} \cdot \pi \cdot r^2} = (4.7 \pm 0.3) \cdot 10^{20}\,\mathrm{m^{-2}s^{-1}} \qquad (3)$$

The measured parameters are:

- Radius of electrochemical cell: $r_0 = 2\,\mathrm{mm}$
- Power of the LED (710 nm) measured through sample holder: $P_{\mathrm{LED}} = (2.36 \pm 0.14)\ \mathrm{mW}$

  (corresponds to an irradiation intensity: $\frac{P_{\mathrm{LED}}}{r^2 \cdot \pi} = (19 \pm 1)\ \mathrm{mW\ cm^{-2}}$)
- Energy per photon: $E_{\mathrm{ph}} = 2.80 \cdot 10^{-19}\ \mathrm{J}$ (710 nm LED, 30 mW/sr, 18°)
- Transmission *T*: 70%, measured via substrate baseline in UV spectra ($=I_{TCB}/I_0$)
- A_{cont} is the *ex situ* estimated via fluorescence microscopy on Ga droplet after contacting a graphite surface: $A_{\mathrm{cont}} = (9.9 \pm 0.6) \cdot 10^2\ \mu\mathrm{m}^2$
- The tunneling current: $I_{\mathrm{t}} = (0.47 \pm 0.18)\ \mathrm{nA}$

Supplementary References

1. Perdew JP, Burke K, Wang Y. Generalized Gradient Approximation for the Exchange-Correlation Hole of a Many-Electron System. *Phys. Rev. B* **54**, 16533-16539 (1996).
2. D. Nečas and P. Klapetek, *Gwyddion: an open-source software for SPM data analysis*, *Cent. Eur. J. Phys.* **10** (1), 181-188 (2012).
3. Nolde F, Qu JQ, Kohl C, Pschirer NG, Reuther E, Müllen K. Synthesis and Modification of Terrylenediimides as High-Performance Fluorescent Dyes. *Chem. Eur. J.* **11**, 3959-3967 (2005).
